# Mortality from malaria in France, 2005 to 2014

**DOI:** 10.2807/1560-7917.ES.2020.25.36.1900579

**Published:** 2020-09-10

**Authors:** Eric Kendjo, Marc Thellier, Harold Noël, Stéphane Jauréguiberry, Alexandra Septfons, Oussama Mouri, Frédérick Gay, Ilhame Tantaoui, Eric Caumes, Sandrine Houzé, Renaud Piarroux, on behalf of the French imported malaria Study group

**Affiliations:** 1 Sorbonne Université, INSERM, Institut Pierre-Louis d’Epidémiologie et de Santé Publique, AP-HP, Laboratoire de Parasitologie, Mycologie, Hôpital Pitié-Salpêtrière, Paris, France; 2 AP-HP, Centre National de Référence du Paludisme, Paris, France; 3 Santé Publique France, Direction des Maladies Infectieuses, Saint-Maurice, France; 4 Sorbonne Université, INSERM, Institut Pierre-Louis d’Epidémiologie et de Santé Publique, AP-HP, Service des Maladies Infectieuses, Hôpital Pitié-Salpêtrière, Paris, France; 5 Université de Paris, MERIT, IRD, Paris, France; 6 Centre National de Référence du Paludisme, Hôpital Bichat, AP-HP, Paris, France; 7 The members of the French imported malaria Study group are acknowledged at the end of this article

**Keywords:** capture-recapture, malaria, *Plasmodium*, travellers, surveillance, FNRCm, France

## Abstract

**Introduction:**

Malaria is a notifiable disease in all European Union and European Economic Area countries except Belgium and France, where only autochthonous malaria is notifiable. Although morbidity caused by malaria has been assessed, little is known about mortality incidence.

**Objective:**

Our aim was to estimate the number of imported malaria-related deaths in hospital in metropolitan France.

**Methods:**

We matched individual deaths reported between 1 January 2005 and 31 December 2014 to the French National Reference Centre for malaria (FNRCm) with malaria-related deaths from two other sources: the French National Registry on medical causes of death and the French national hospital discharge database. A capture–recapture method with log-linear modelling was used. Age, sex and place of death stratification were applied to remove heterogeneity.

**Results:**

The estimated malaria-related deaths in metropolitan France during the study period were 205 (95% confidence interval (CI): 191–219). The annual mean number of malaria-related deaths was estimated at 21 (95% CI: 19–22). The FNRCm malaria-related deaths surveillance had a 38% sensitivity (95% CI: 32–44). Among 161 in-hospital individual malaria-related deaths reported from three data sources, the sex ratio (male to female) was 2.6. Median age of the patients was 57 years, ranging from 1 to 89 years.

**Conclusion:**

The pertinent finding of this report is that malaria-related death records were significantly more complete than case records. Therefore, data comparison of imported malaria morbidity and mortality between countries should imperatively be assessed using standard indicators weighted according to the completeness of health surveillance systems.

## Introduction

In 2018, malaria ranked 16th among the leading causes of death [1], with an estimated 228 million cases and more than 405,000 deaths worldwide [2]. The World Health Organization European Region is the first in the world to have achieved interruption of autochthonous malaria transmission at the end of the 20th century. However, malaria remains a threat for travellers to endemic areas. It is a notifiable disease in all European Union (EU) and European Economic Area (EEA) countries except Belgium and France, where only autochthonous malaria is notifiable. Based on data retrieved from The European Surveillance System (TESSy), 30 EU/EEA countries reported 8,393 confirmed malaria cases in 2017 [3]. France reported the highest number of cases (n = 2,712), followed by the United Kingdom (UK) (n = 1,777) and Germany (n = 956) [3]. Despite a diminution of cases and death in endemic areas, the 2017 figures for the EU/EEA were 27% higher than in 2013 (n = 6,614) [3]. Most cases of imported malaria in France and the UK are linked to travel to West Africa [4].

Among 4,874 confirmed malaria cases in the 30 EU/EEA countries for whom the *Plasmodium* species was available, 3,989 (81.8%) were infected with *P. falciparum*. The case fatality was 1.5% among 1,220 cases with *P. falciparum* malaria and a known outcome [3]. However, even in countries where malaria is a mandatorily reported disease, there is significant under-reporting of cases and deaths. Thus, the completeness of the collection of cases was estimated at 21.2% for the period 2001 to 2011 in Portugal, 32.9% for 2016 in Portugal (after changing the case reporting system), 40.2% in 1996 in the Netherlands and 56% between July 2003 and December 2004 in the UK [5-8]. In addition, as has been shown in Portugal, the completeness of the collection may be different for cases (21.2%) and for deaths (26.5%) [6]. 

In metropolitan France, using a homogenised definition for severe malaria cases, a recent study of the French National Reference Centre for Malaria (FNRCm) showed an increase in the proportion of severe malaria cases from 8.9% in 1996 to 16.7% in 2016 [9]. Surprisingly, over the same period, the number of deaths from malaria reported to FNRCm remained stable, ranging from five to 10 each year, with a case fatality rate of ca four per 1,000 [9,10]. The sensitivity of the FNRCm surveillance system for cases varied from 50% to 55% [11]. However, at present, the sensitivity of the FNRCm surveillance systems is unknown for individual deaths. Under-notification hides the real burden of malaria morbidity and mortality and negatively affects indicators for adequate malaria control. Therefore, the main objective of this study was to estimate the number of malaria-related deaths in hospitals in France.

## Methods

## Study design

A cross-sectional analysis was performed on mortality data using capture–recapture methods to estimate the number of individual deaths caused by all malaria species in metropolitan France from 2005 to 2014 [12]. The date period was chosen because modifications were made to the coding mode on the CépiDc database in 2000 and because accurate data for the period 2015 and 2016 were not available.

### Data sources

Three data sources are available to capture malaria deaths in France: the French National Registry on Medical Causes of Death (CépiDc) [13], the French National Hospital Discharge Database (PMSI) [14], and the FNRCm [15]. These sources are fully digitalised and include individual-based data of malaria-related deaths in France. Data from these sources are used for public health monitoring.

#### The French National Registry on Medical Causes of Death

The CépiDc has been managed by the French National Institute of Health and Medical Research (Inserm)’s public health programme since 1968. The main objective of the CépiDc is the yearly production and analysis of national statistics of the medical causes of death in metropolitan France and overseas departments. This source helps authorities acquire a comprehensive understanding of the strengths and weaknesses of their mortality statistics systems. It also derives demographic and epidemiological measures that are needed in national planning in the health sector [13]. In France, death certification is mandatory and necessarily performed by a physician. The underlying cause of death is defined as the disease or injury that initiates the succession of morbid events leading to death [2]. The CépiDc contains one underlying cause of death (main cause) and more consecutive contributory causes of death (associated causes). Only some basic administrative information (date of birth, sex, place of birth, place of usual residence, date of death, place of death, and socio-professional category) is collected from death certificates. Since 2000, diagnoses reported to the CépiDc have been coded according to the International Classification of Diseases, 10th revision (ICD-10) [16,17].

#### The French National Hospital Discharge Database

The PMSI collects information on every hospital stay in France. Each hospital discharge report, corresponding to a hospital stay, is described according to the following items: reasons for hospitalisation (principal diagnosis) and related medical conditions (associated diagnoses) by ICD-10 code, length of hospital stay and characteristics of the patient (age, sex, place of hospitalisation and residence, health event/mortality) [14,16].

#### The French National Reference Centre for Malaria

The FNRCm is a sentinel network of ca 100 hospitals that fully reports malaria cases to a secured database and sends blood samples to reference laboratories based on voluntary participation. The mission of the FNRCm is to monitor the morbidity and mortality of imported malaria, investigate autochthonous cases and assess the chemosensitivity of *P. falciparum* isolates. It provides health authorities with real-time information on malaria-specific events, such as increasing cases from particular transmission areas, emergence of resistance to available drugs or re-emergence of malaria in French non-endemic areas or territories [18-20]. Clinical and epidemiological data are collected for each individual diagnosed with malaria.

### Case definitions

Malaria-related death was defined as a death occurring in an acute infectious context with the presence of malaria severity criteria combined with the absence of another cause likely to be life-threatening in the short term. Severe malaria cases were defined using the 2007 revision of the 1999 consensus conference [21,22].

### Study population strategy

To carry out this work, the first step was to merge the databases from two sources that aim at exhaustive collection of deaths and their causes (CépiDC and PMSI) and from a source with a partial and targeted collection (FNRCm) and remove any duplicates. As the CépiDC and PMSI databases were not independent (Supplementary Table S1), a merger was carried out between them (CépiDC–PMSI). Owing to the great heterogeneity in the collection and coding of the causes of death in the CépiDC and PMSI databases, our second concern was to rule out deaths potentially linked to a cause other than malaria.

### Merging cases

The merging of the databases was carried out using a unique key derived from sex, the month of death, the year of death and the place of death. For deduplication and validation of cases, we used the variable ‘age at death’, which was common to all sources of data. When the age difference of matched cases was equal to zero, the merging was validated. Otherwise, couples of paired patients with differences in the age at death (i.e. differences not equal to zero) were manually checked for validation by comparing all available data.

### Selection of malaria-related deaths

Each report from selected patients was carefully checked in parallel by two parasitologists (MT and RP) and one specialist in infectious diseases (SJ) to classify the cases according to the most likely causes of death (malaria but also suicide, trauma, cancer, lymphoma, knife or firearm wound, stroke, etc). To accomplish this first step, the experts met to rule on the discordant cases. If the three experts did not agree that malaria was the most likely cause of death, the case was excluded.

### Statistical analysis

#### Capture–recapture methods

These methods are used to estimate the size of a population when a census may be infeasible or impossible to conduct. A portion of the population is captured, marked and released. Later, another portion will be captured and the number of marked individuals within the sample is counted.

Dependence between sources was assessed through two-by-two contingency table analysis and odds ratios with 95% confidence intervals (CI) [23,24].

#### Sensitivity analysis: log-linear models [25]

To validate our methodology, log-linear models incorporating interaction terms to account for dependencies between the three overlapping sources were used to predict the number of unobserved deaths and, hence, the total number of deaths in the study population. The significance of each interaction was assessed using likelihood ratio statistics, and goodness of fit of the model was measured by the deviance. The Akaike information criterion (AIC) and Bayesian information criterion (BIC) were used for the model selection.

Stratification was made for sex, age at death (coded as < 50 years-old and ≥ 50 years-old), and place of death to take into account the heterogeneity between the data sources. The place of death was categorised as Paris region vs other regions since Paris represented 37% (49/132) of hospitals participating in the FNRCm network during the study period.

#### Estimating the sensitivity of each source using the capture–recapture method

The sensitivity of each source is a measure of how well the source performs in finding cases. The sensitivity was estimated by dividing the number of cases found using the case-finding procedure by the estimate of the total number of cases in the study population found by the capture–recapture method.

#### Representativeness of the FNRCm network

The representativeness of the FNRCm surveillance system was assessed by comparing the age, sex and administrative region structure of malaria-related deaths with those from CépiDc combined with PMSI over the same period. We assumed that malaria-related deaths selected from these sources were representative of the total malaria-related deaths in France.

Statistical analyses were performed using JMP pro, version 13.1 (SAS Institute). Log-linear models were performed using Stata, version 15.0 (StataCorp., College Station, United States). A difference was considered significant when p < 0.05. All reported p values were two-tailed.

### Ethical statement

Approval of the study was obtained from the Ethics Committee for Biomedical Research of the French National Agency for Medicines and Health Products (ANSM) in declaration number ID RCB: 2018-A02469–46 with a waiver of specific consent by the patients. Permission was sought and granted to access these data on 11 August 2017, by the Ethics Committee of the French Institute for Public Health Surveillance (SPF). Data collection and storage by the FNRCm were approved by the French National Commission for Data Protection and Liberties (CNIL) under declaration number 1223103. All data from the FNRCm sources were de-identified by removing names and scrambling all other personal identifiers.

## Results

**Figure 1:**
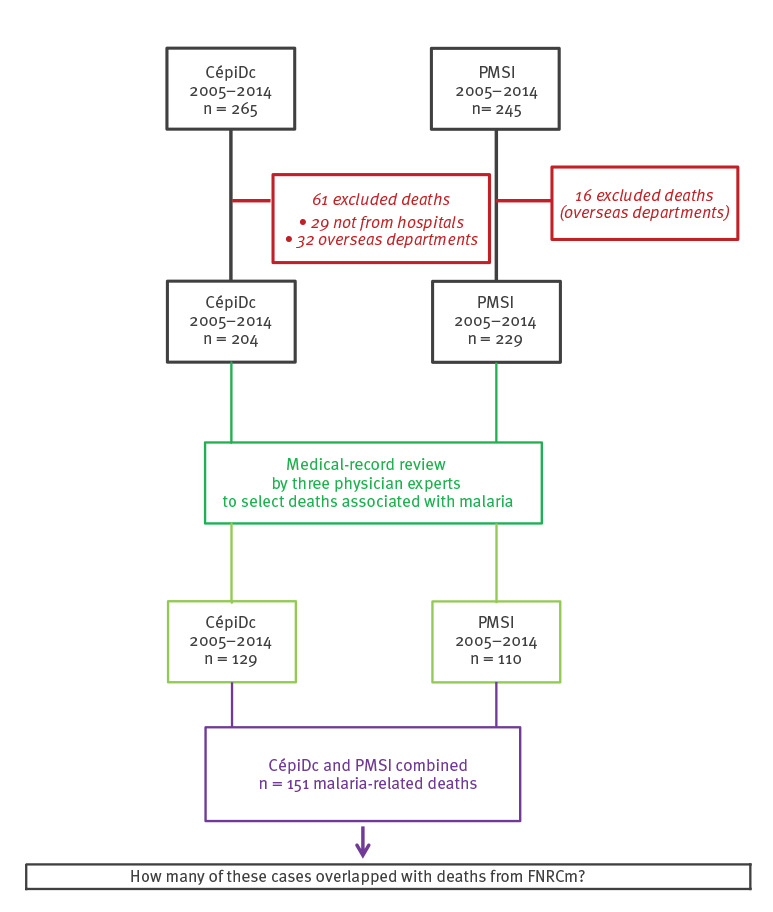
Flowchart of malaria-related deaths from two sources, CépiDc and PMSI, metropolitan France, 2005–2014 (n = 151)

**Figure 2:**
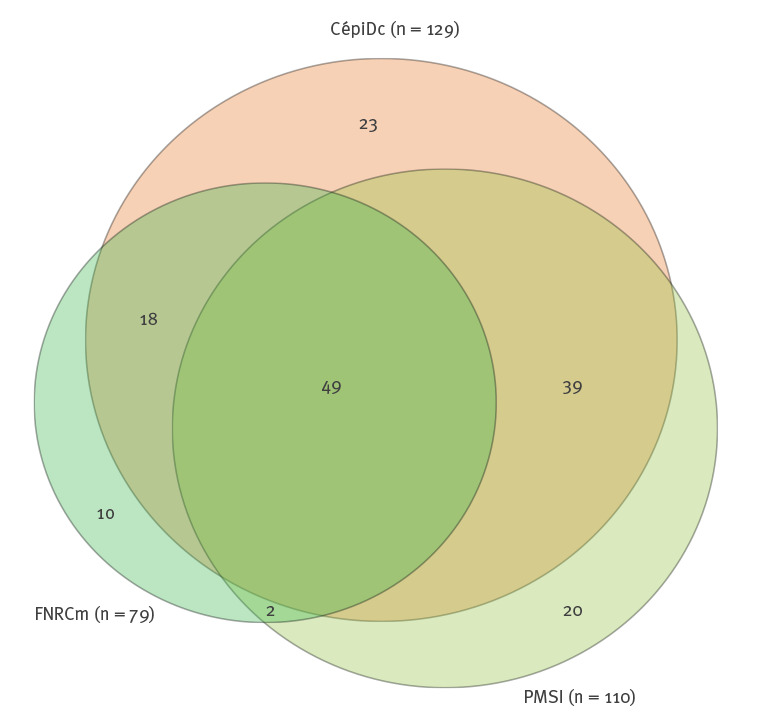
Venn diagram over the distribution of individual deaths, showing overlap and unique cases for three data sources according to the experts’ classification of cause of death, metropolitan France, 2005–2014 (n = 161)

From 2005 to 2014, the experts reclassified 151 deaths from CépiDc and PMSI as associated with malaria, of which 129 were registered in CépiDc and 110 in PMSI (Figure 1). For the same period, 79 deaths were selected from the FNRCm, of which 69 overlapped with both CépiDc and PMSI (Figure 2). After removing duplicate cases, the overall number of malaria-related deaths resulting from the combination of both data sources was 161.

CépiDc: French National Registry on Medical Causes of Death; FNRCm: French National Reference Centre for Malaria; PMSI: French National Hospital Discharge Database (Programme de Médicalisation des Systèmes d’Information).

CépiDc: French National Registry on Medical Causes of Death; FNRCm: French National Reference Centre for Malaria; PMSI: French National Hospital Discharge Database (Programme de Médicalisation des Systèmes d’Information).

### Interdependence between CépiDc–PMSI and FNRCm

Prior analysis had shown a dependence between Cépidc and PMSI (Supplementary Table S1); however, the two-by-two contingency table analysis suggested independence between the combined CépiDc–PMSI and FNRCm, with OR = 0.72 (95% CI: 0.38–1.37).

### Two-by-two capture–recapture models (CépiDc–PMSI vs FNRCm)

Using the two-by-two capture–recapture method without taking into account heterogeneity factors, the FNRCm malaria-related deaths surveillance had a 46% (95% CI: 44–54) sensitivity, leading to an estimated number of in-hospital malaria-related deaths in metropolitan France of 172 (95% CI: 165–184), of which 11 (95% CI: 8–15) were not captured by either source (Supplementary Figure S1).

### Sensitivity analysis: Log-linear models

Estimates based on the log-linear models are shown in Table 1. The final model with the smallest AIC values, corresponding to a better adapted model, was the one with interaction between CépiDc and FNRCm**, **leading to 172 (95% CI: 165–184) estimated deaths.

**Table 1:** Log-linear-based estimates of the number of individual deaths from *Plasmodium* malaria according to the physician experts’ classification, metropolitan France, 2005–2014 (n = 161)

Model	DoF	G^2^	p	AIC	BIC	Unknown deaths	n estimated	95% CI
Main effects only^a^ (CépiDc, PMSI, FNRCm)	3	20.58	<0.00	14.58	14.74	7	168	163–176
Main effects + (CépiDc^a^–PMSI)	4	17.72	<0.00	13.72	13.83	11	172	164–187
Main effects + (CépiDc^a^–FNRCm)	4	13.97	<0.00	9.97	10.08	11	172	165–184
Main effects + (PMSI^a^–FNRCm)	4	20.53	<0.00	16.53	16.64	6	167	162–177
Main effects + (CépiDc^a^–PMSI, PMSI^a^–FNRCm)	5	17.65	<0.00	15.65	15.71	12	173	164–194
Main effects + (CépiDc^a^–FNRCm, PMSI^a^–FNRCm)	5	13.93	<0.00	11.93	11.99	11	172	164–187
Main effects + (CépiDc^a–^PMSI, CépiDc^a^–FNRCm, PMSI^a^–FNRCm)	6	0	1	0	0	160	321	191–1,383

AIC: Akaike information criterion; BIC: Bayesian information criterion; CépiDc: French National Registry on Medical Causes of Death; CI: confidence interval; DoF: degree of freedom; FNRCm: French National Reference Centre for Malaria; G^2^: likelihood ratio G² hypothesis test; PMSI: French National Hospital Discharge Database (Programme de Médicalisation des Systèmes d’Information).

^a^ Main effects only is the model with CépiDc, PMSI and FNRCm, without interaction terms.

Models include interaction terms involved in the adjustment of estimates for dependence among sources.

This number was similar to the previous estimation made by the two-by-two capture–recapture method (Supplementary Figure S1). However, when accounting for age as a heterogeneity factor, the overall estimated number of individual malaria-related deaths was substantially higher, 205 (95% CI: 191–219), consequently reducing the sensitivity of the FNRCm network to 38% (95% CI: 32%–44%). This number was particularly high for the age group ≥ 50 years with 140 deaths (95% CI: 133–147). In contrast, the variables sex and place of death were not found to be heterogeneity factors in this study (Table 2). Given these results, the mean annual estimated number of deaths caused by malaria in France was 21 (95% CI: 19–22).

**Table 2:** Estimates of the total number of malaria-related deaths stratified by sex, age in years and place of death, metropolitan France, 2005–2014 (n = 161)

	CépiDc–PMSI	FNRCm	(CépiDc–PMSI) + FNRCm	FNRCm alone	CépiDc–PMSI alone	Var(n)	X?	n estimated	95% CI
**Sex**									
Female	41	27	23	4	18	6.551	3	48	43–53
Male	110	52	46	6	64	22.566	8	124	115–134
**Place of death**									
Paris region	60	28	25	3	35	11.29	4	67	61–74
Other regions	91	51	44	7	47	17.925	7	105	97–114
**Age (years)**									
< 50	48	30	24	6	24	13.46	11	65	58–72
≥ 50	103	49	45	4	58	12.849	33	140	133–147

CépiDc: French National Registry on Medical Causes of Death; CI: confidence interval; FNRCm: French National Reference Centre for Malaria; X?: unknown number of deaths; PMSI: French National Hospital Discharge Database (Programme de Médicalisation des Systèmes d’Information); Var(n): variance.

### General characteristics of the 161 deaths captured by the PMSI, CépiDc and FNRCm

The malaria-related deaths collected by the PMSI, CépiDc and FNRCm accounted for 78.5% (95% CI: 72.4–83.6) of the estimated number of deaths caused by malaria in metropolitan France from 2005 to 2014. The sex ratio (male to female) was 2.6. The median age of the patients was 57 years (interquartile range: 44–64), ranging from 1 to 89 years. The Île-de-France region reported 41.6% (n = 67) of the deaths (Supplementary Table S2).

### Representativeness of the FNRCm network

Compared with the three sources combined (CépiDc–PMSI–FNRCm), the proportion of individual malaria-related deaths was smaller in the FNRCm for the age group ≥ 50 years (62.0% vs 66.5%) and for men (65.8% vs 72.1%), but the differences were not statistically significant. The geographical distribution of the number of deaths caused by malaria was significantly different between the FNRCm and the three sources combined. Malaria-related deaths reported to FNRCm were mostly from the regions Pays de la Loire, Provence-Alpes-Côte d'Azur and Nouvelle Aquitaine (Supplementary Table S3).

## Discussion

This study was the first attempt to estimate the total number of deaths caused by malaria in metropolitan France. From 2005 to 2014, the total number of malaria-related deaths in hospitals estimated by capture–recapture methods was 205 (95% CI: 191­–219), which was notably different from the 79 deaths reported to the FNRCm. Consequently, only 38% (95% CI: 32–44) of malaria-related deaths were reported to the FNRCm, leading to an overall estimate of 21 (95% CI: 19–22) deaths per year*.* Given the completeness of our surveillance system, which captures between 50% and 53% of cases, our finding was surprising [9,11]. The FNRCm surveillance system is less sensitive in the capture of deaths than the capture of cases. This discrepancy suggests that deaths occur more frequently in hospitals not included in the FNRCm network. However, because information on population structure is lacking, we are not able to conclude on differences in the population structure of people with malaria reported to the FNRCm by the network of participating hospitals (mainly teaching hospitals or inter-regional/departmental hospitals in large cities) and other hospitals that see fewer cases of malaria infection. 

The strengths of this study include the ability of our surveillance system to estimate the number of deaths from malaria and the stability in the morbidity and mortality trend previously established by the FNRCm network over time [2]. Comparison of malaria morbidity and mortality data within and between countries using standard indicators weighted according to the estimated completeness of health surveillance systems is imperative to allow reliable evaluation of malaria surveillance programmes and to homogenise disease surveillance policies in all EU countries. In the UK and the Netherlands, where malaria is a notifiable disease, the case reporting systems have 56% and 40.2% sensitivity, respectively. However, this statistic is not available for malaria deaths [7,26]. Consequently, there could be a substantial difference between the collection of cases and deaths, as reported in our study. In contrast to the high completeness of malaria cases reported in France, the sensitivity of the system in Portugal was higher for malaria deaths (26.5%) than for cases (21.2%) [6].

Once the data were analysed for age, sex and place of death of the deceased, there were more estimated malaria deaths among people aged 50 years and older than in those younger than 50 years: 140 deaths (95% CI: 133–147) vs 65 deaths (95% CI: 58–72). A study conducted in intensive care units in France has shown that a 10-year increase in age was associated with a 72% increase in the odds of hospital death [27]. Similarly, other studies on reported malaria deaths demonstrated that age was a risk factor for predicting death in patients [28-32].

Our study highlights a marked difference between male and female patients who died of malaria in metropolitan France (sex ratio: 2.6). This seems surprising since evidence in the literature suggests that, given equal exposure and apart from the specific case of pregnancy, adult men and women are equally vulnerable to malaria infection with no differences regarding specific mortality in malaria endemic areas [33,34]. However, similar findings have already been reported in the literature regarding reported malaria in high-income countries (sex ratio: 1.5–5.0) [27,28,32,35,36]. Differences between the sexes in the incidence and severity of infection may be related to genetic and physiological constitutions [37] or to differences in exposures or immunity [38]. Although a causal relationship has not been established, many elements confirm this sex difference. For instance, studies have shown that female sex is independently associated with receiving pretravel health information and with increased concern about travel stressors [39,40]. Based on data from 58,908 patients in the GeoSentinel networks, Schlagenhauf et al. reported that women were significantly more likely to have sought pretravel advice (odds ratio (OR) = 1.28; 95% CI: 1.23–1.32), were proportionately less likely to have febrile illnesses (OR = 0.15; 95% CI: 0.10–0.21) or vector-borne diseases (such as malaria) (OR = 0.46; 95% CI: 0.41–0.51) and were less likely to be hospitalised (OR = 0.45; 95% CI: 0.42–0.49) [38]. A difference in the behaviour of men and women with respect to malaria prevention or care is suspected, but the role of a genetic difference is not formally excluded. The lack of difference between the sexes observed in endemic areas could be explained by the protective effect of immunity in adults [41]. Therefore, there is a real need for future travel medicine research to address sex-specific intervention strategies and differential susceptibility to malaria.

Our study also showed that the estimated number of malaria-related deaths reported from the Paris region represented 38% of the total malaria-related deaths (Table 2). This can be explained by the fact that most cases were diagnosed in the Île-de-France region (n = 25,647; 56.6%), the French administrative region surrounding Paris, with 12,082,144 inhabitants in January 2015 and 37% of hospitals participating in the FNRCm network.

### Limitations

The first limitation concerns the accuracy of data from CépiDc and PMSI. Clinical information for some patients was not sufficient to conclude whether the death was caused by the malaria. Indeed, in the capture–recapture method, inclusion of cases with an erroneous diagnosis leads to underestimation of the real number of cases (because false positive cases cannot be recaptured). Consequently, if the experts had been less strict on the definition of malaria-related deaths, the inclusion of non-malaria deaths would have resulted in an underestimation of the completeness of the sources and therefore, the estimation of individual malaria-related deaths. Conversely, not including all the true cases in the first capture step does not induce estimation bias since the final result is related to the proportion of recaptured cases and not to the absolute number of recaptured cases. Indeed, according to fundamental assumptions of capture–recapture methods, i.e. independence of the sources and equal probability of individual cases being captured within any source regardless of the number of deaths selected, each death from the CépiDc–PMSI had the same probability of being recaptured by the FNRCm. Reducing the estimated number of malaria deaths selected should therefore not affect the estimates of completeness of the data sources and, consequently, the estimation of the number of deaths caused by malaria. Owing to the delays in making data available from the national databases of CépiDc and the PMSI, our study covered data until the end 2014. However, since 2014, the network of FNRCm correspondents has remained stable, and even though the first-line drug for the treatment of severe malaria changed in 2011 (artesunate replaced quinine), there was no significant impact on mortality, which was already very low in our hospital network [42]. Moreover, in view of the FNRCm data on deaths and CFR during the period 2015 to 2018, there was no significant variation in the CFR compared with the period 2005 to 2014 (Supplementary Table S4). Another limitation of this study is that it exclusively concerned patients who died in the hospital. Patients who died at home were not included in either the PMSI or FNRCm, making an estimate of this number not feasible by this method. However, the CépiDC can indicate an order of magnitude for this number since, in the study period, 29 deaths that occurred outside the hospital were declared (ca three per year). A range close to reality should therefore be between four and five additional deaths per year.

## Conclusion

The pertinent finding in this report is the significant difference in the completeness of malaria-related deaths compared with cases. Therefore, data comparison of malaria morbidity and mortality between countries, especially in TESSy, should be assessed by using standard indicators weighted according to the completeness of countries’ health surveillance systems for malaria cases and deaths. It is also appropriate, in the FNRCm, to improve the exhaustiveness of the collection of malaria-related deaths that frequently occur outside our network. One of the approaches to improve this exhaustiveness might be to involve further laboratories/hospitals in smaller cities that carry out the diagnosis of malaria. The current modifications made to the structure of the FNRCm network should be validated and routinely combined to improve the completeness of malaria death reporting and information used to conduct timely preventive measures by public health authorities.

## Supplementary Data

428000 Supplement
