# Authors’ correction for Euro Surveill. 2020;25(36)

**DOI:** 10.2807/1560-7917.ES.2020.25.38.200924c

**Published:** 2020-09-24

**Authors:** Eurosurveillance editorial team

**Affiliations:** 1 European Centre for Disease Prevention and Control (ECDC), Stockholm, Sweden

In the article ‘Mortality from malaria in France, 2005 to 2014’ by Kendjo et al. published on 10 September 2020, At publication, the Abstract originally stated that malaria-related death records were “more complete” than case records, while it should have read “less complete”. This error was corrected on request of the authors on 24 September 2020.

