# Supplementary material for: Mortality from malaria in France, 2005 to 2014
Source: Euro Surveill. 2020 Sep 10;25(36):1900579. doi: 10.2807/1560-7917.ES.2020.25.36.1900579 (PMC7502900; doi:10.2807/1560-7917.ES.2020.25.36.1900579)
Supplement: Supplement [file 19-00579_KENDJO_Supplement.pdf]

Supplementary material

*This supplementary material is hosted by Eurosurveillance as supporting information alongside the article [**Mortality from malaria in France, 2005 to 2014**], on behalf of the authors, who remain responsible for the accuracy and appropriateness of the content. The same standards for ethics, copyright, attributions and permissions as for the article apply. Supplements are not edited by Eurosurveillance and the journal is not responsible for the maintenance of any links or email addresses provided therein.*

Supplementary Table S1. Interdependence between sources prior expert selection, Metropolitan France, 2005-2014.

Contingency Analysis of CépiDc By PMSI

Count<br>Col %	Yes	No	Total
Yes	99<br>48.53	130<br>90.91	229
No	105<br>51.47	13<br>9.09	118
Total	204	143	347

Odds Ratio

Odds Ratio	Lower 95%	Upper 95%
0.094286	0.050075	0.177528

Contingency Analysis of CépiDc By FNRCm

Count<br>Col %	Yes	No	Total
Yes	72<br>35.29	16<br>11.19	88
No	132<br>64.71	127<br>88.81	259
Total	204	143	347

Odds Ratio

Odds Ratio	Lower 95%	Upper 95%
4.329545	2.390488	7.84148

Contingency Analysis of PMSI By FNRCm

Count<br>Col %	Yes	No	Total
Yes	57<br>24.89	31<br>26.27	88
No	172<br>75.11	87<br>73.73	259
Total	229	118	347

Odds Ratio

Odds Ratio	Lower 95%	Upper 95%
0.930045	0.559751	1.545301

Supplementary Table S2. General characteristics of deaths by data sources, Metropolitan France, 2005-2014.

	CépiDc		PMSI		FNRCm		Total	
	**N**	**%**	**N**	**%**	**N**	**%**	**N**	**%**
**Gender**								
Female	37	28.7%	31	28.2%	27	34.2%	45	28.0%
Male	92	71.3%	79	71.8%	52	65.8%	116	72.0%
Total	129	100.0%	110	100.0%	79	100.0%	161	100.0%
**Age (yrs)**								
< 5	1	0.8%	0	0.0%	2	2.5%	2	1.2%
5-9	2	1.6%	2	1.8%	1	1.3%	2	1.2%
10-14	2	1.6%	2	1.8%	0	0.0%	2	1.2%
15-19	4	3.1%	4	3.6%	3	3.8%	4	2.5%
20-24	3	2.3%	2	1.8%	1	1.3%	3	1.9%
25-29	5	3.9%	4	3.6%	4	5.1%	7	4.3%
30-34	7	5.4%	4	3.6%	5	6.3%	8	5.0%
35-39	6	4.7%	4	3.6%	4	5.1%	7	4.3%
40-44	6	4.7%	3	2.7%	4	5.1%	7	4.3%
45-49	9	7.0%	6	5.5%	6	7.6%	12	7.5%
50-54	19	14.7%	13	11.8%	10	12.7%	20	12.4%
55-59	20	15.5%	17	15.5%	14	17.7%	23	14.3%
60-64	22	17.1%	21	19.1%	15	19.0%	29	18.0%
65-69	10	7.8%	10	9.1%	4	5.1%	12	7.5%
70-74	5	3.9%	7	6.4%	2	2.5%	9	5.6%
>= 75	8	6.2%	11	10.0%	4	5.1%	14	8.7%
Total	129	100.0%	110	100.0%	79	100.0%	161	100.0%
**Place of death**								
Auvergne-Rhône-Alpes	12	9.3%	10	9.1%	4	5.1%	13	8.1%
Bourgogne-Franche-Comté	1	0.8%	2	1.8%	1	1.3%	2	1.2%
Bretagne	3	2.3%	3	2.7%	2	2.5%	3	1.9%
Centre-Val de Loire	3	2.3%	3	2.7%	3	3.8%	5	3.1%
Grand-Est	10	7.8%	8	7.3%	6	7.6%	11	6.8%
Hauts-de-France	4	3.1%	4	3.6%	3	3.8%	5	3.1%
Île-de-France*	48	37.2%	42	38.2%	29	36.7%	67	41.6%
Normandie	5	3.9%	5	4.5%	0	0.0%	6	3.7%
Nouvelle-Aquitaine	17	13.2%	15	13.6%	12	15.2%	20	12.4%
Occitanie	9	7.0%	6	5.5%	4	5.1%	11	6.8%
Pays de la Loire	4	3.1%	4	3.6%	4	5.1%	4	2.5%
Provence-Alpes-Côte d'Azur	13	10.1%	8	7.3%	11	13.9%	14	8.7%
Total	129	100.0%	110	100.0%	79	100.0%	161	100.0%

**Paris region*

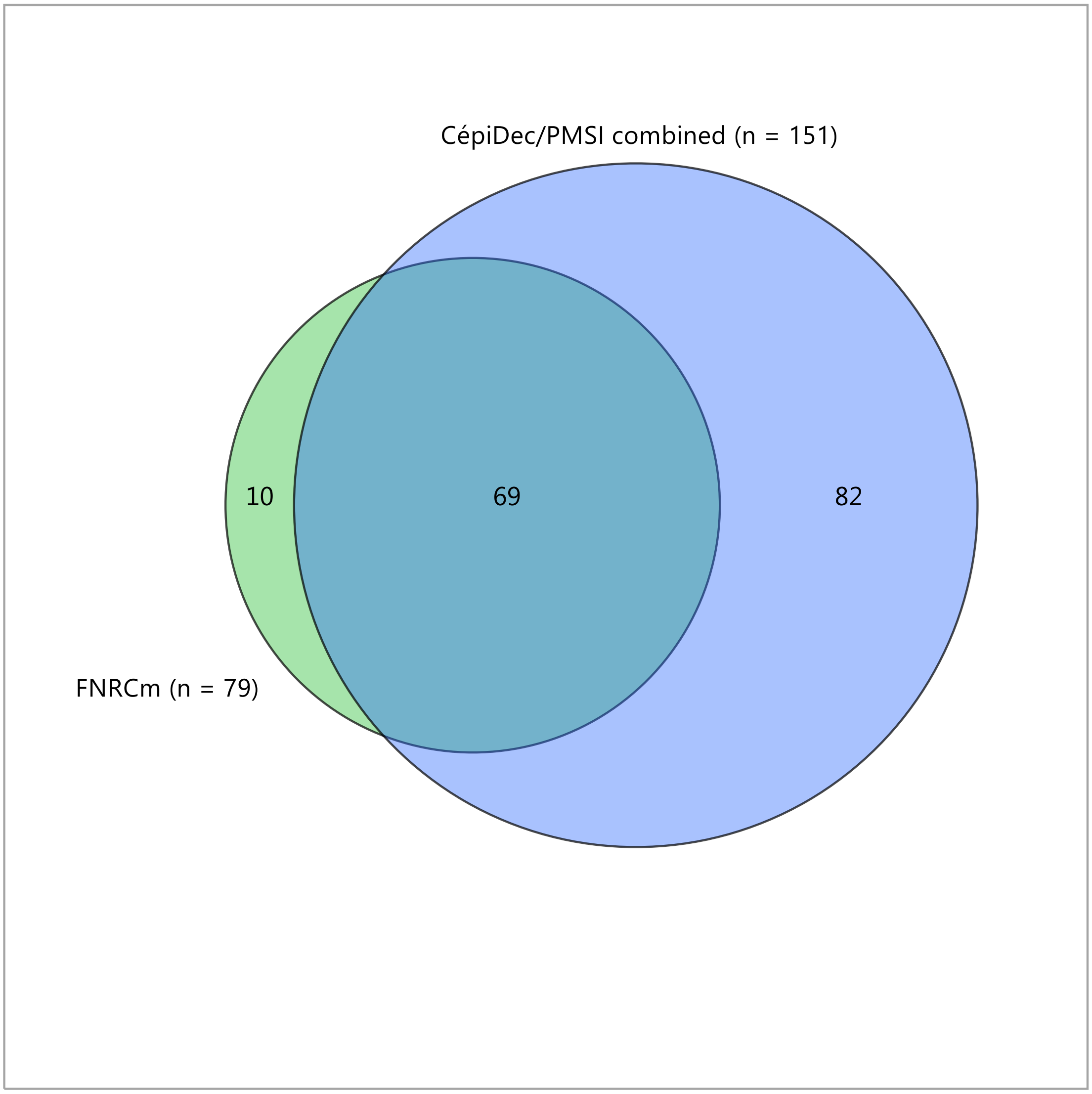

Supplementary Figure S1. The Venn diagram above depicts the distribution of the number of individual deaths in terms of the overlap as well as the unique cases for CépiDc-PMSI combined and the French National Registry on medical causes of death (FNRCm) data sources, metropolitan France, 2005-2014.

Supplementary Table S3. General characteristics of reported malaria-related deaths between CépiDc-PMSI-*FNRCm* combined and the *French National Reference Centre for malaria (FNRCm)* data sources, metropolitan France, 2005-2014.

**Variables**	**CépiDc-PMSI- *FNRCm***		***FNRCm***		**Difference (%FNRCm; %CépiDc-PMSI- FNRCm)**
	**N**	**%**	**N**	**%**	**%**
**Age**					
≥ 50 years	*107*	*66.5%*	*49*	*62.0%*	*-4.5%*
**Gender**					
Male	*116*	*72.1%*	*52*	*65.8%*	*-6.3%*
**Administrative regions**					
Auvergne-Rhône-Alpes	*13*	*8.3%*	*4*	*5.1%*	*-3.2%*
Bourgogne-Franche-Comté	*2*	*1.3%*	*1*	*1.3%*	*0.0%*
Bretagne	*3*	*1.9%*	*2*	*2.5%*	*0.7%*
Centre-Val de Loire	*5*	*3.2%*	*3*	*3.8%*	*0.7%*
Grand-Est	*11*	*7.0%*	*6*	*7.6%*	*0.7%*
Hauts-de-France	*5*	*3.2%*	*3*	*3.8%*	*0.7%*
*Île-de-France**	*63*	*40.1%*	*29*	*36.7%*	*-2.4%*
Normandie	*6*	*3.8%*	*0*	*0.0%*	*-3.8%*
Nouvelle-Aquitaine	*20*	*12.7%*	*12*	*15.2%*	*2.6%*
Occitanie	*11*	*7.0%*	*4*	*5.1%*	*-1.9%*
Pays de la Loire	*4*	*2.5%*	*4*	*5.1%*	*2.6%*
Provence-Alpes-Côte d'Azur	*14*	*8.9%*	*11*	*13.9%*	*5.2%*

*Paris region

Supplementary Table S4. Imported malaria deaths and CFR from FNRCm, 2005-2014 and 2015-2018.

Years	**Deceased**	**Cases**	**CFR %**
2005	9	2,683	0.34
2006	10	2,754	0.36
2007	6	2,203	0.27
2008	9	2,245	0.40
2009	7	2,248	0.31
2010	9	2,467	0.36
2011	4	2,002	0.20
2012	13	1,889	0.69
2013	11	2,233	0.49
2014	11	2,374	0.46
2005-2014	**89**	**23,098**	**0.39**
2015	12	2,543	0.47
2016	5	2,484	0.20
2017	13	2,739	0.47
2018	11	2,842	0.39
2015-2018	**41**	**10,608**	**0.39**
